# Diversity and Ecophysiology of the Genus OLB8 and Other Abundant Uncultured *Saprospiraceae* Genera in Global Wastewater Treatment Systems

**DOI:** 10.3389/fmicb.2022.917553

**Published:** 2022-07-08

**Authors:** Zivile Kondrotaite, Laura C. Valk, Francesca Petriglieri, Caitlin Singleton, Marta Nierychlo, Morten K. D. Dueholm, Per H. Nielsen

**Affiliations:** Department of Chemistry and Bioscience, Center of Microbial Communities, Aalborg University, Aalborg, Denmark

**Keywords:** wastewater, activated sludge, *Saprospiraceae*, OLB8, high-quality MAGs, FISH–Raman, MiDAS

## Abstract

The *Saprospiraceae* family within the phylum Bacteroidota is commonly present and highly abundant in wastewater treatment plants (WWTPs) worldwide, but little is known about its role. In this study, we used MiDAS 4 global survey with samples from 30 countries to analyze the abundance and distribution of members of *Saprospiraceae*. Phylogenomics were used to delineate five new genera from a set of 31 high-quality metagenome-assembled genomes from Danish WWTPs. Newly designed probes for fluorescence *in situ* hybridization (FISH) revealed rod-shaped morphologies for all genera analyzed, including OLB8, present mostly inside the activated sludge flocs. The genomes revealed potential metabolic capabilities for the degradation of polysaccharides, proteins, and other complex molecules; partial denitrification; and storage of intracellular polymers (glycogen, polyphosphate, and polyhydroxyalkanoates). FISH in combination with Raman microspectroscopy confirmed the presence of intracellular glycogen in *Candidatus* Brachybacter, *Candidatus* Parvibacillus calidus (both from the former genus OLB8), and *Candidatus* Opimibacter, and the presence of polyhydroxyalkanoates in *Candidatus* Defluviibacterium haderslevense and *Candidatus* Vicinibacter. These results provide the first overview of the most abundant novel *Saprospiraceae* genera present in WWTPs across the world and their potential involvement in nutrient removal and the degradation of macromolecules.

## Introduction

Wastewater treatment is an essential process that protects humans and the environment from anthropogenic waste, and currently, it is shifting toward resource recovery and sustainability (Loosdrecht et al., 2016; Qasim, 2017). Microorganisms are the most important actors in wastewater treatment systems, with their capabilities to degrade diverse organic substances and to cycle elements such as nitrogen, phosphorus, and carbon (Daims et al., 2006b; Wen et al., 2010; Zaborowska et al., 2019). However, most of the microorganisms involved in these processes are still poorly described.

The phylum Bacteroidota (former Bacteroidetes) is one of the most abundant and diverse lineages in wastewater treatment plants (WWTPs), comprising bacteria with various metabolisms (Thomas et al., 2011; Kwon et al., 2019; Al Ali et al., 2020). Their main role in WWTPs has been described in relation to the degradation of complex organic substances, such as proteins, starch, cellulose, and fibers (Yang et al., 2017). In addition, members of this phylum are able to degrade dead cells and exopolysaccharides into simpler organic compounds, such as lactate and ethanol, which can then be utilized by other members of the community (Al Ali et al., 2020).

*Saprospiraceae* is the most abundant family in this phylum, with representatives in WWTPs across the world (Muszyński et al., 2015; Yang et al., 2015, 2017; Al Ali et al., 2020; Cao et al., 2020). Several isolates have been obtained from different water environments (Hosoya et al., 2006; Chen et al., 2014; McIlroy and Nielsen, 2014) and are reported to have variable morphology, spanning from filamentous to rods of different lengths, with some genera forming helical gliding filaments (McIlroy and Nielsen, 2014). Interestingly, filamentous *Saprospiraceae* such as *Haliscomenobacter* can occasionally be involved in bulking problems in WWTPs (Xu et al., 2018). However, the most abundant genera, such as OLB8, are widely found in different environments such as activated sludge (Al Ali et al., 2020; Aqeel and Liss, 2020; Seshan et al., 2021) and anammox reactors (Speth et al., 2016). The first metagenome-assembled genome (MAG) of the genus OLB8 was recovered from partial nitrification anammox bioreactors, where the bacterium was known to function as an aerobe heterotroph (Speth et al., 2016). Members of the genus OLB8 were also found in high abundance in reactors with polyphosphate-accumulating organism (PAO)-enriched biomass (Arumugam et al., 2019), bioreactors with a high ratio of food to microorganisms (Roy et al., 2020), reactors with increased concentration of ammonia (Aqeel and Liss, 2020), deammonification reactors (Chini et al., 2020), and also in reactors related to degradation of aromatic compounds (Seshan et al., 2021). Over the years, OLB8 bacteria were speculated to be involved in phosphorus and nitrogen removal in addition to the degradation of different carbon compounds in WWTPs, but their function was never fully resolved. Most of the *Saprospiraceae* genera are still uncharacterized, with unknown functions; thus, further analysis is needed to fully understand their role in WWTPs.

Since only a few *Saprospiraceae* isolates exist from the WWTP ecosystem, the function most of these microorganisms can only be inferred using metabolic models derived from annotated genomes MAGs, preferably high-quality (HQ) MAGs (Parks et al., 2017; McDaniel et al., 2021; Singleton et al., 2021). While in 2015, only five genomes were available for *Saprospiraceae* (McIlroy and Nielsen, 2014), recent effort has raised that number to the 134 representative public genomes currently available in the GTDB (R06-RS202) (Parks et al., 2020). However, extensive metabolic reconstruction is still lacking. *In situ* studies have shown a strong metabolic capacity to decompose proteins, lipids, and other macromolecules into simple compounds (Luo et al., 2020). For example, the genus *Candidatus* Epiflobacter plays an important role in protein degradation by the production of extracellular enzymes (Xia et al., 2008). Moreover, members of *Saprospiraceae* are often found in microbial communities involved in nitrogen removal (Sun et al., 2012; Cao et al., 2020), indicating their potential role as nitrogen-transforming bacteria, although this has to be confirmed experimentally.

The taxonomy of Bacteroidota has undergone several changes in the last decade, resulting in inconsistent classification, with major differences depending on the reference database used (Hahnke et al., 2016; Parks et al., 2020; Schoch et al., 2020). MiDAS, a comprehensive and ecosystem-specific full-length 16 rRNA gene reference database of microbes found in Danish and global WWTPs, was established 5 years ago and is still expanding (Karst et al., 2018; Nierychlo et al., 2020; Dueholm et al., 2022). It is a powerful tool that provides taxonomy information for all bacteria and is useful for both community composition analysis and for the design and re-evaluation of oligonucleotide probes for fluorescence *in situ* hybridization (FISH). The FISH technique is important for the visualization of microorganisms of interest and also essential for the validation of genomic information when combined with other *in situ* techniques (Nielsen et al., 2009).

In this study, we investigate the diversity of 5 highly abundant novel genera within the *Saprospiraceae* in activated sludge WWTPs worldwide. The morphology and spatial arrangement of the *Saprospiraceae* species were studied with newly designed genus- and family-level FISH probes. The FISH probes were also used to investigate the presence of intracellular storage polymers with FISH–Raman microspectroscopy. A detailed metabolic reconstruction was made using the recently published ecosystem-specific HQ MAGs (Singleton et al., 2021). By combining the global MiDAS 4 16S rRNA gene reference (Dueholm et al., 2022) and the HQ MAG database with FISH techniques, we obtained a comprehensive understanding of the diversity and function of abundant members of *Saprospiraceae* in the activated sludge ecosystem.

## Methods

### Sampling, Amplicon Sequencing, and Bioinformatics Analysis

Sampling of full-scale plants across the world was coordinated by the MiDAS Global Consortium and carried out within the Global MiDAS project (Dueholm et al., 2022). Global activated sludge samples from the process tanks were preserved in RNA later and shipped to Aalborg University with cooling elements.

Sequencing of full-length 16S rRNA genes was performed as previously described by Karst et al. (2018). After sequencing, data for bioinformatics analysis was performed as explained in Dueholm et al. (2020, 2022). Raw and assembled sequencing data are available at the NCBI SRA database BioProject ID: PRJNA728873. Data were analyzed using R v4.1.1 (R Core Team, 2020) and R studio software v1.4.1717 (RStudio Team, 2020) and visualized with ampvis2 v2.7.10 (Andersen et al., 2018) and ggplot v3.2.1 (Wickham, 2016) packages. The Kruskal–Wallis test (Kruskal-Wallis Test, 2008) was used to determine statistically significant differences in genus abundance across different groupings.

### Phylogenetic Analysis, Fluorescence *in situ* Hybridization (FISH) Probe Design, and Evaluation

Phylogenetic analysis of 16S rRNA gene sequences and design of FISH probes for *Saprospiraceae* were performed using ARB software v6.0.6 (Ludwig, 2004). The phylogenetic maximum likelihood tree was constructed based on comparative analysis of aligned 16S rRNA gene sequences, retrieved from MiDAS 4 (Dueholm et al., 2022), using the GTR method and a 1,000 replicates bootstrap analysis. Coverage and specificity of the FISH probes were evaluated and validated *in silico* with mathFISH software (Yilmaz et al., 2011) for hybridization efficiencies of target and potentially weak non-target matches. When needed, unlabeled competitor, and helper probes were designed. All probes were purchased from Biomers (Ulm, Germany), labeled with cyanine 3 (Cy3), cyanine 5 (Cy5), Atto 532, Atto 565, Atto 594, and Atto 488 fluorochromes.

### FISH, Quantitative FISH (qFISH), and Raman Microspectroscopy

The biomass collected from full-scale activated sludge WWTPs was fixed with 50% ethanol (final volume) or 4% PFA (final volume), and stored at −20°C for further analysis. FISH was performed as described by Nielsen et al. (2009). Optimal formamide concentrations for each new FISH probe (Supplementary Table S1) were determined after performing hybridization at different formamide concentrations in the range of 0–70% (with 5% increments). The intensity of 50 cells was measured using ImageJ software (Schneider et al., 2012). EUBmix (Amann et al., 1990; Daims et al., 1999) was used as a general probe to target all bacteria. Microscopic analysis was performed with either an Axioskop epifluorescence microscope (Car Zeiss, Germany) equipped with a LEICA DFC7000 T CCD camera or a white light laser confocal microscope (Leica TCS SP8 X, Leica Microsystems, Germany). qFISH was performed for each genus-specific probe, targeting bacteria of interest, labeled with Cy3 dye, and used together with EUBmix labeled with Cy5 as a universal probe. Analysis was performed on a set of 30 images captured using a 63 x microscope objective on a white light laser confocal microscope (Leica TCS SP8 X, Leica Microsystems, Germany) and analyzed with DAIME v2.2.2 software (Daims et al., 2006a). Raman microspectroscopy was applied in combination with FISH. This approach allows the detection of general cellular components, such as nucleic acids, membrane lipids or proteins, as well as intracellular storage polymers, such as polyphosphate (poly-P), glycogen, and polyhydroxyalkanoates (PHA), as previously described (Fernando et al., 2019).

### MAG Identification, Annotation, and Metabolic Reconstruction

Genomes from *Saprospiraceae* were identified in a set of 1,083 HQ MAGs recovered from Danish WWTPs (Singleton et al., 2021). Taxonomic classification at the phylogenetic level in relation to the genome taxonomy database and maximum likelihood placement was conducted using GTDB-Tk v1.4.1 (Chaumeil et al., 2019) (Refseq release 95) “classify.” Pyani v0.2.11 (Pritchard et al., 2016) was used to determine the species (using a 95% average nucleotide identity (ANIb) cutoff) (Singleton et al., 2021) and genus (using a 75–77% ANI cutoff). To determine MAG completeness and contamination statistics, CheckM v1.1.2 (Parks et al., 2015) “lineage_wf” was used. Multiple sequence alignments of 120 concatenated single copy proteins, trimmed to ~5,000 amino acids, produced by GTDB-Tk were used as input for IQ-TREE v2.0 (Nguyen et al., 2015) to create a maximum likelihood tree using the WAG+G model and 100-replicates bootstrap analysis. *Saprospiraceae* genomes were selected for inclusion in the bootstrapped tree based on the GTDB-Tk v1.5.0 tree and the paraphyletic clade incorporating all genomes of interest. The resulting bootstrapped tree of *Saprospiraceae* was further examined and rooted in ARB v6.0.3 (Ludwig, 2004), and ITOL v6 (Letunic and Bork, 2021) was used for tree visualization with final aesthetic changes made in Inkscape v1.0.1. Members of the genera *Lewinella, Haliscomenobacter*, and *Saprospira* were used as an outgroup.

MAGs were annotated using EnrichM v0.5.0 (github.com/geronimp/enrichM) “annotate” with default settings to blast the MAG proteins against the uniref100 database (Suzek et al., 2015) annotated with KEGG (Kanehisa, 2000). Orthology (KO) numbers (EnrichM database v10). In addition, the MAGs were uploaded to the “MicroScope Microbial Genome Annotation & Analysis Platform” (Vallenet et al., 2019) to examine the gene synteny and cross-validate KO annotations found using EnrichM. The presence of a metabolic pathway was assumed when the full set of genes, defined by the KEGG module and “MicroScope Microbial Genome Annotation & Analysis Platform,” were predicted. In this study, pathways are considered present if 100% of the genes in the KEGG or custom module were encoded.

## Results and Discussion

### Abundance and Distribution of *Saprospiraceae* in WWTPs Worldwide

**Figure 1:**
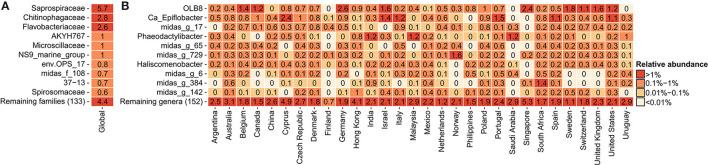
Global average relative read abundance in 929 activated sludge samples: **(A)** 10 most abundant families within Bacteroidota and **(B)** 10 most abundant genera within *Saprospiraceae*.

In order to determine the most abundant genera of *Saprospiraceae* and to investigate their diversity and key factors correlating with their global presence, a detailed analysis was performed on the V1-V3 amplicon data obtained from the recent Global MiDAS survey (Dueholm et al., 2022), comprising 929 samples from 30 countries. Bacteroidota was the second most abundant phylum in the WWTPs (Supplementary Figure S1), with *Saprospiraceae* being the most abundant family (Figure 1A), in accordance with previous observations (Yang et al., 2015, 2017; Al Ali et al., 2020; Cao et al., 2020; Luo et al., 2020). *Saprospiraceae* was very diverse and comprised more than 162 different genera based on MiDAS 4, which included the known *Ca*. Epiflobacter (Xia et al., 2008), *Phaeodactylibacter* (Chen et al., 2014), and *Haliscomenobacter* (van Veen et al., 1973), but the majority of the genera were putative with assigned placeholder names by AutoTax (Dueholm et al., 2020) and undescribed. The high diversity within the family and the difference in taxonomic classification using different databases suggest that the classification and taxonomy of *Saprospiraceae* need revision. In this study, we decided to focus on the three most abundant and novel genera defined by MiDAS 4.7.1 with the provisional names OLB8, midas_g_17, and midas_g_65. In most of the countries, these three genera co-existed in the same plants, with the highest global abundances recorded mostly in European countries, such as Germany (2.6% relative read abundance) for OLB8, Italy (0.9%) for midas_g_17, and Portugal (0.6) for midas_g_65 (Figure 1B).

**Figure 2:**
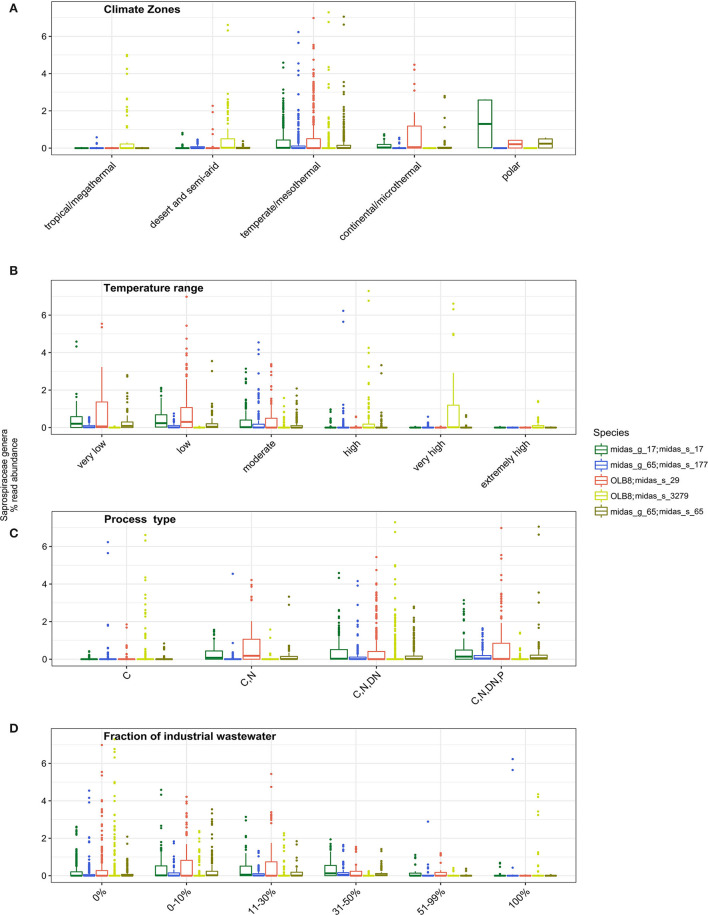
Occurrence of different *Saprospiraceae* species across the world in relation to **(A)** different climate zone groupings (tropical/megathermal climate, 29 plants; dry (desert and semi-arid) climate, 48 plants; temperate/mesothermal climate, 368 plants; continental/microthermal climate, 24 plants; polar climate, 2 plants) (Kruskal–Wallis for all species *p* < 0.05; Supplementary Table S3); **(B)** global average relative read abundance of highly abundant species of *Saprospiraceae* in different temperature ranges analyzed in process tanks [very low (1–10.0°C, 43 plants), low (10.1–15.0°C, 96 plants), moderate (15.1–20.0°C, 112 plants), high (20.1–25°C, 73 plants), very high (25.1–30.0°C, 48 plants), extremely high (30.1–38.0°C, 32 plants)] (Kruskal–Wallis for all species *p* < 0.05; Supplementary Table S3); **(C)** process type (C−113 plants; C, N, 48 plants; C, N, DN, 208 plants, C, N, DN, P, 111 plants), (C, carbon removal; N, nitrification; DN, denitrification; P, biological P removal), (Kruskal–Wallis for all species *p* < 0.05; Supplementary Table S3) and **(D)** In WWTPs with different fractions of industrial wastewater (0%, 169 plants; 0–10%, 105 plants; 11–30%, 67 plants; 31–50%, 41 plants; 51–99%, 20 plants; 100%, 40 plants) (Kruskal–Wallis for all species *p* < 0.05; Supplementary Table S3).

More in-depth analyses of the five most abundant species in genera OLB8 (midas_s_29, midas_s_3279), midas_g_17 (midas_s_17), and midas_g_65 (midas_s_65, midas_s_177) were carried out to give insights into factors affecting their occurrence across the world (Figure 2). Some studies have shown a seasonal distribution of members belonging to S*aprospiraceae*, with a potential preference for lower temperatures (Muszyński et al., 2015; Xu et al., 2018; Luo et al., 2020). We applied Köppen–Geiger climate classification (Kottek et al., 2006) to determine differences in the abundance distribution within different climate zones. While midas_s_3279 had higher abundance in tropical/megathermal, dry (desert and semi-arid), and temperate/mesothermal climates, midas_s_65, midas_s_17, and midas_s_29 seemed to be most abundant in temperate/mesothermal climate, continental/microthermal, and polar climates, featuring more humid and colder climates (Figure 2A and Supplementary Table S2).

We observed a strong effect of temperature in process tanks of WWTPs on the abundance of the members of *Saprospiraceae*. The highest abundance was noticed at very low, low, and moderate temperatures for midas_s_29, midas_s_17, midas_s_65, and midas_s_177, while at the higher process tank temperature ranges, these members were present at a very low abundance (Figure 2B). Unlike all other members, midas_s_3279 was mostly abundant in WWTPs with very high temperatures (25.1–30°C) (Figure 2B). In addition, midas_s_3279 was observed in high relative abundance in deammonification reactors (up to 15%) (Supplementary Figure S2) with low oxygen and high temperatures.

The process design influenced the abundance of most species, with highest abundances occurring in more advanced plants with biological N and P removal (Figure 2C). This is supported by previous observations of highly abundant members of *Saprospiraceae* in plants performing N and P removal with anaerobic and anoxic stages (Sun et al., 2012; Yang et al., 2015). The fraction of industrial wastewater in the influent (here given as the COD fraction) (Figure 2D) was also shown as an important and statistically significant factor influencing the abundance of all the species, based on Kruskal–Wallis statistical analysis. All species were found mainly in municipal plants with low or no influx of industrial wastewater (<30%) (Figure 2D) in agreement with other studies (Yang et al., 2017; Chi et al., 2018; Luo et al., 2020; Schambeck et al., 2020).

### Phylogenetic Analysis and FISH Probe Coverage of *Saprospiraceae*

**Figure 3:**
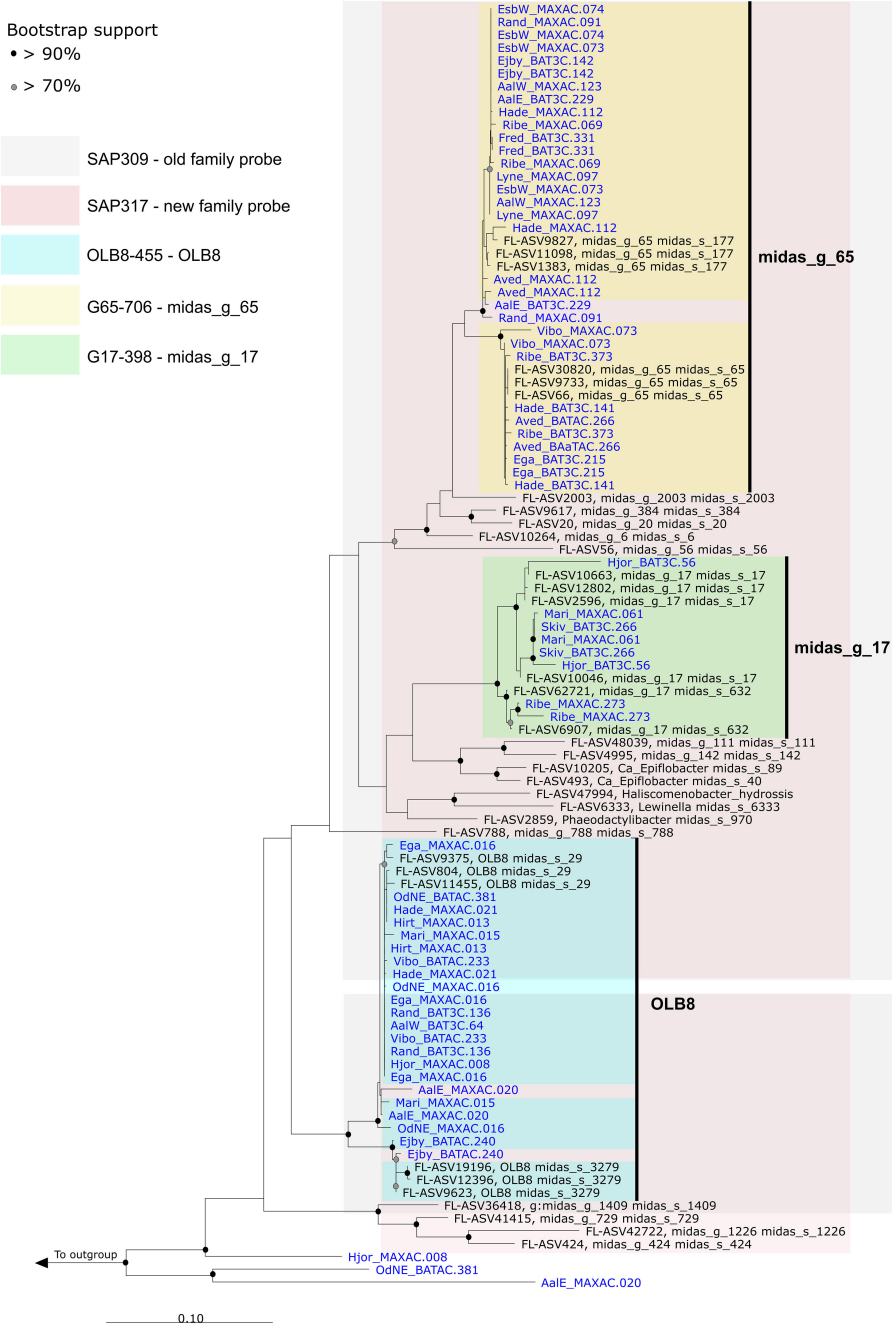
Maximum-likelihood (PhyML) phylogenetic tree based on 16S rRNA genes of the *Saprospiraceae* including full-length sequences from MiDAS 4 (black) and MAG (blue). Colors indicate the coverage of FISH probes designed in this study. Bootstrap values from 1000 re-samplings are indicated for branches with >70% (gray circles) and >90% (black circles) support. The scale bar represents substitutions per nucleotide base.

16S rRNA gene-based phylogenetic analysis of *Saprospiraceae* was performed using full-length sequences obtained from MiDAS 4 (Dueholm et al., 2022) and from 32 HQ MAGs classified as the genera OLB8, midas_g_17, and midas_g_65, recovered from 23 Danish WWTPs (Singleton et al., 2021; Figure 3).

The first FISH probes targeting *Saprospiraceae* were designed over 27 years ago (Wagner et al., 1994), and to date, there are only 21 FISH probes available to target 11 specific genera, species, or clusters within this family (Ginige et al., 2004; McIlroy and Nielsen, 2014). Among these, the widely applied family-level probe SAP-309 (Schauer and Hahn, 2005) was tested *in silico* using the MiDAS 4 reference (Figure 3). Even though the test indicated a good specificity, the use of this probe as a general probe cannot be recommended due to insufficient coverage (Supplementary Table S1). A new family-level probe SAP-317 was designed and optimized for a better coverage of *Saprospiraceae* present in WWTPs (Figure 3 and Supplementary Table S1). Furthermore, three additional probes were designed using the MiDAS 4 taxonomy, with competitors and helper probes, targeting the genera OLB8, midas_g_17, and midas_g_65 (Figure 3 and Supplementary Table S1). When applied to full-scale activated sludge biomass, all three probes exclusively hybridized to short, thick rods found predominantly as single cells inside the flocs and rarely attached to other bacteria (Supplementary Figure S3).

Quantitative FISH analysis with all three new probes showed that the biovolume fractions of the biomass were lower than those of the relative read abundance by amplicon sequencing using V1-V3 primers (Supplementary Table S4). It could be due to the presence of 2–3 copies of the 16S rRNA gene within each MAG (Figure 3 and Supplementary Data File 1) or due to the different sizes of cells within the activated sludge biomass. Primers can have a considerable impact on the abundance recorded by the amplicon sequencing (Dueholm et al., 2022), so we compared the average global abundance obtained using the two commonly applied primer sets, V1–V3 and V4. The average abundance of members of *Saprospiraceae* (genus level) was similar between both primers sets (Supplementary Figure S4).

### Genome Recovery and Phylogenomic Analysis Reveal Several Novel Genera

Overall, 32 HQ MAGs representing different genera in *Saprospiraceae* (Supplementary Table S5) from 23 Danish WWTPs (Singleton et al., 2021) were investigated. Completeness and contamination of all MAGs analyzed ranged between 91.88–99.01% and 0–4.95%, respectively, and they all contained 2–3 copies of the 16S rRNA gene (Supplementary Data File 1).

**Figure 4:**
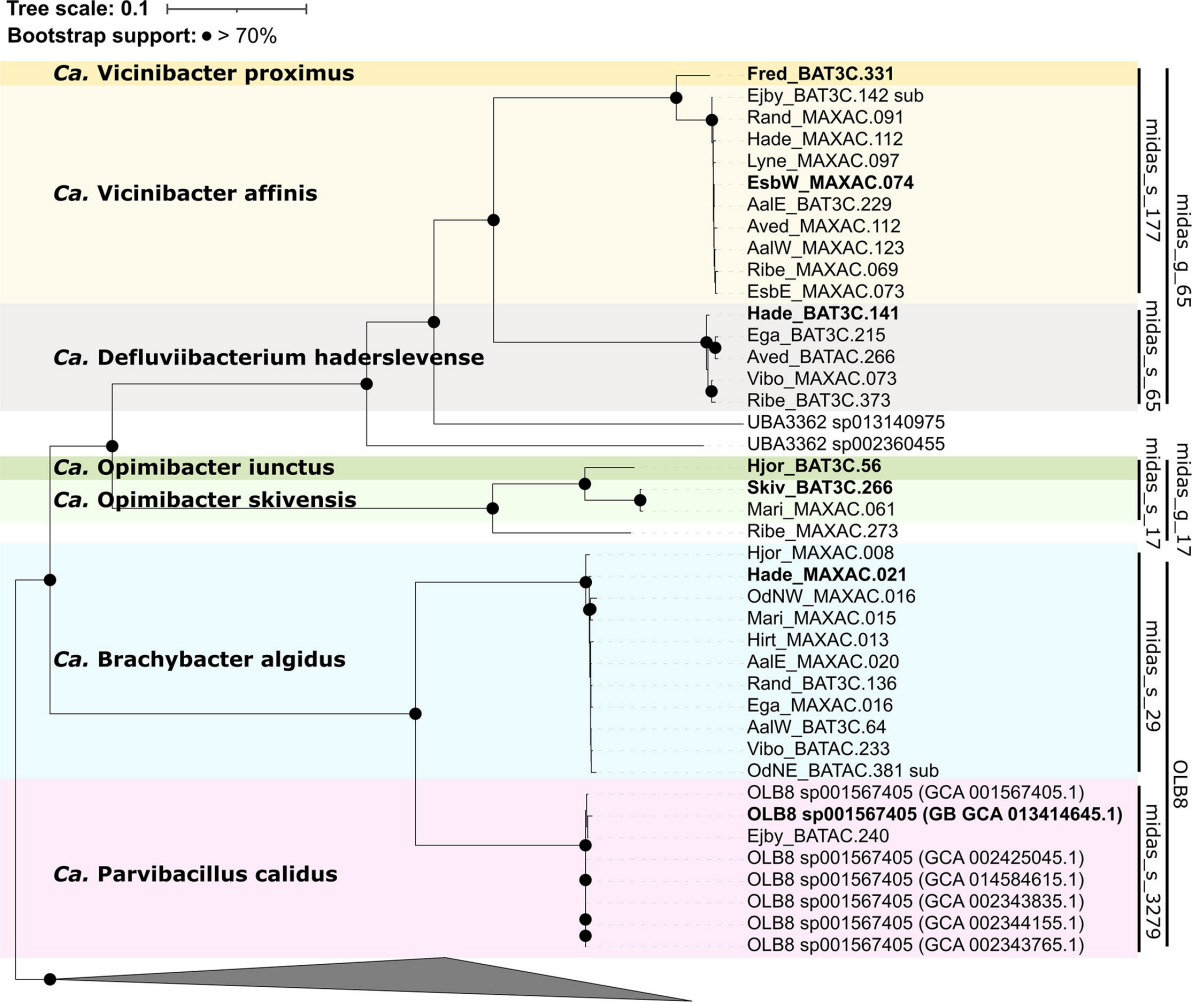
Maximum likelihood genome tree created from the concatenated alignment of 120 single-copy marker gene proteins trimmed to 5,000 amino acids using the GTDB-Tk v1.5.0. Branches with bootstraps support >70% are indicated by black dots. Species representatives are in bold. The genera *Lewinella, Haliscomenobacter*, and *Saprospira* were used as the outgroup.

Phylogenomic analysis using the GTDB-Tk (Figure 4) showed that all OLB8 midas_s_29 MAGs grouped with the OLB8 genus as one novel species (>95% ANI) (Chaumeil et al., 2019), and one MAG representing midas_s_3279, which grouped with OLB8 sp001567405 (GCA_001567405.1) species. The analysis additionally showed that all MAGs from midas_g_65 clustered together with the UBA3362 genus (GCA_002360455.1), while the MAGs from midas_g_17 represented a novel genus and did not cluster with any of the known *Saprospiraceae* genera.

To determine the taxonomic rank classification, analysis of the full genome average nucleotide identity (ANI) was performed with proposed boundaries for genus (75–77%) and species (>95%) levels (Barco et al., 2020; Parks et al., 2020). The analysis showed that the HQ MAGs represented six novel genera and seven novel species (Figure 4), contrary to previous 16S rRNA gene-based taxonomic analysis. Based on the phylogenomic analysis, the species midas_s_29 (>98% ANI) and midas_s_3279 (>99% ANI), both belonging to the genus OLB8 according to MiDAS and GTDB-Tk taxonomy, represent two different genera (<75% ANI clustering) (Supplementary Figure S5). In addition, based on ANI clustering, 16 MAGs representing midas_g_65 clustered in two different genera and three different species, grouping together next to the close relative UBA3362 sp002360455 (Parks et al., 2017) but representing a separate genus (<75% ANI) (Supplementary Figure S5). Furthermore, midas_s_17 and midas_s_632, both belonging to the MiDAS-defined genus midas_g_17 based on ANI clustering analysis, appeared to be two novel genera with three novel species (Supplementary Figure S5). Due to the very low abundance of midas_s_632 in activated sludge in both Danish and global WWTPs, we excluded it from further analysis. Overall, the clustering of analyzed members did not show any major differences between the 16S rRNA gene and the genome tree (Figures 3, 4).

For the two new genera encompassing midas_s_29 and midas_s_3279, respectively, we propose the names *Candidatus* Brachybacter algidus (Supplementary Table S6) and *Candidatus* Parvibacillus calidus (Supplementary Table S7). For further analysis, seven additional HQ MAGs from the public GTDB (OLB8 sp001567405) were included in the metabolic reconstruction for better representation of *Ca*. P. calidus. For midas_s_65, we propose the name *Candidatus* Defluciibacterium haderslevense (Supplementary Table S8), and for midas_s_177, which represents two different species based on ANI analysis, we propose the names *Candidatus* Vicinibacter proximus (Supplementary Table S9) and *Candidatus* Vicinibacter affinis (Supplementary Table S10). For midas_s_17, which includes two species, we propose the names *Candidatus* Opimibacter skivensis (Supplementary Table S11) and *Candidatus* Opimibacter iunctus (Supplementary Table S12).

### Metabolic Reconstruction of Novel *Saprospiraceae* Genera

**Figure 5:**
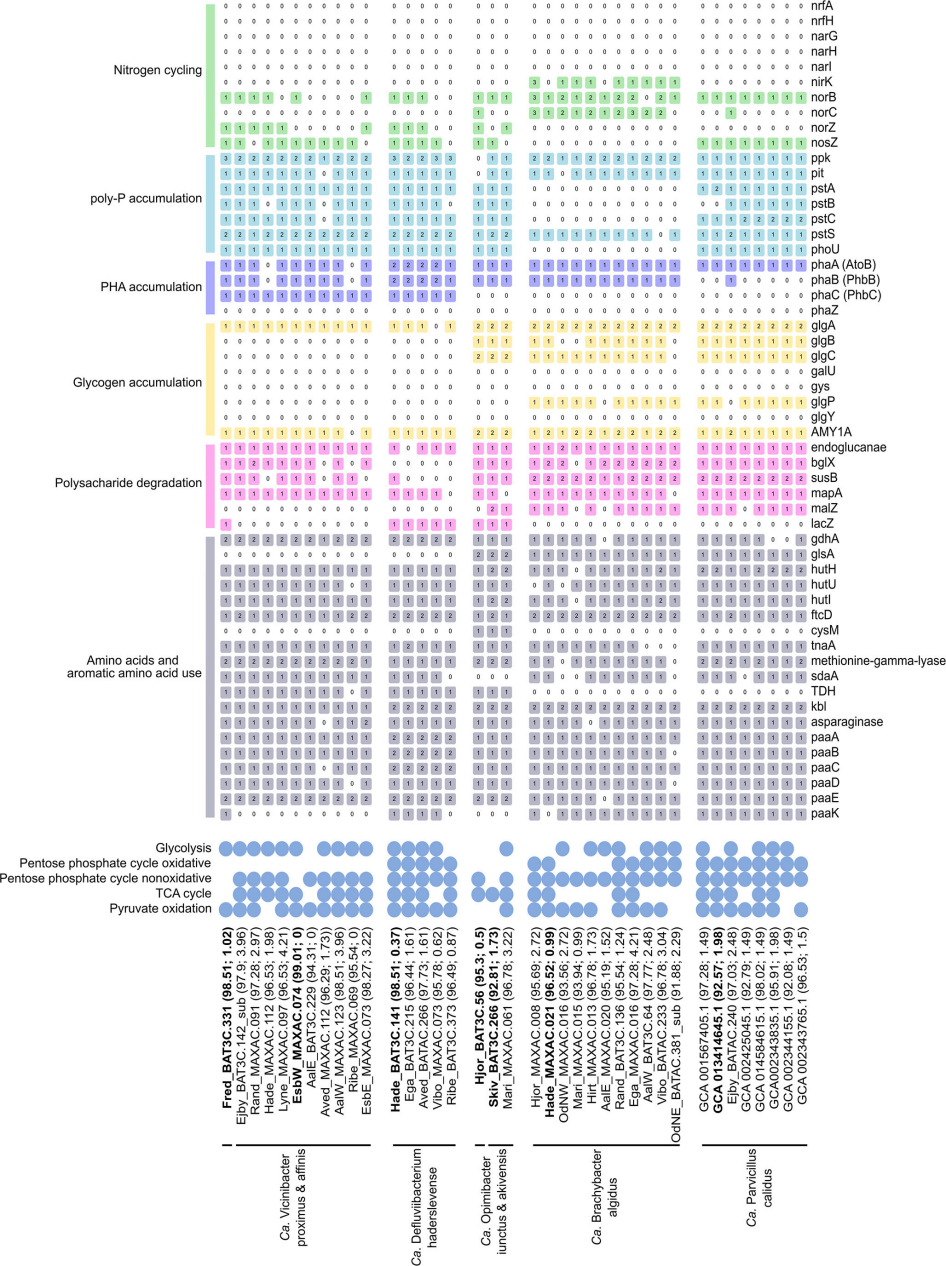
Basic functional potential of the *Ca*. Vicinibacter, *Ca*. Defluviibacterium haderslevense, *Ca*. Opimibacter, *Ca*. Brachybacter algidus, and *Ca*. Parvibacillus calidus. The number in colored boxes represents gene copy number. The gene list follows the progression in the text. For the full list of gene names and associated KO numbers, see Supplementary data 3. The genomes are ordered as in the genome tree in Figure 4, with their genome completeness and contamination indicated within the parentheses. Species representatives are in bold.

In this section, we have investigated the metabolic potential of key wastewater treatment processes: carbon degradation, nitrogen transformation, polyphosphate accumulation, and fermentation (Figure 5 and Supplementary Data Files 3, 4). The five genera analyzed showed diverse metabolisms, with a general heterotrophic lifestyle.

The potential for glucose utilization through the full Embden–Meyerhof–Parnas (EMP) pathway (KEGG module: M00001) was encoded in MAGs from *Ca*. B. algidus, *Ca*. P. calidus, and *Ca*. O. skivensis. Even though the non-oxidative pentose phosphate pathway (M00007) was predicted in all analyzed species, the full pentose phosphate pathway (M00004) was only predicted in *Ca*. B. algidus and *Ca*. P. calidus. A complete TCA cycle (M00009) was found in MAGs from all analyzed species (Figure 5). The cytochrome c oxidase (M00155) pathway was also identified in all species; in addition, the aerobic respiration (cytochrome c) pathway was present in MAGs from *Ca*. B. algidus (Supplementary Data File 4).

In previous studies of Bacteroidota, it was shown that some genera are able to utilize polysaccharides as the carbon source (Xia et al., 2008; Terrapon et al., 2018; Lapébie et al., 2019). The genes for endoglucanase and beta-glucosidase (*blgX*), responsible for cellulose degradation to glucose, were found in most of the MAGs (Figure 5). In addition, genes responsible for starch/glycan (*susB*) degradation to glucose (Kitamura et al., 2008) were present in all the MAGs, except those representing *Ca*. D. haderslevense and *Ca*. O. skivensis (Figure 5). Maltose phosphorylase (*mapA*), which is used for maltose degradation (Gopal et al., 2010), was present in all five genera. In 13 MAGs representing *Ca*. O. skivensis, *Ca*. B. algidus, and *Ca*. P. calidus, the gene *malZ*, responsible for sucrose degradation, was found. In addition, the *lacZ* gene, which divides lactose into glucose and galactose, was found in all MAGs from *Ca*. Opimibacter, *Ca*. D. haderslevense, and *Ca*. V. proximus (Figure 5). The capability to degrade polysaccharides was indicated by the presence of *susC/D* homologs, which predict polysaccharide utilization loci (PULs), present in many Bacteroidota genomes. In addition, the phylum-exclusive type IX secretion system (T9SS), which is highly associated with polysaccharide degradation when PUL-encoded enzymes are secreted by T9SS (McKee et al., 2021), was found in all five genera (Supplementary Data File 3). These findings agree with studies of many isolates of Bacteroidota having high numbers of extracellular carbohydrate active enzymes, indicating a potential to degrade various high-molecular weight polysaccharides (McKee et al., 2021).

In addition, it has been observed that *Ca*. Epiflobacter is able to utilize amino acids as a carbon source under oxic, anoxic, and short-term anaerobic conditions (Xia et al., 2008). In all MAGs from all five genera genes were found for the degradation of several amino acids, such as glutamate (*glutamate dehydrogenase*), glutamine (*glutaminase*), histidine (*hutHUI, ftcD, fold*), L-cysteine (*cysM/tnaA/CTH*), methionine (*methionine-gamma-lyase*), L-serine (*sdaA*), threonine (*TDH, kbl*), tryptophan (*tnaA*), alanine (*ald*), and asparagine (*asparaginase*) (Figure 5).

Fermentation is another important growth metabolism for bacteria present in WWTPs (Kong et al., 2008). According to genome annotation, all five newly described genera have, to some extent, the possibility to ferment (Supplementary Data File 3). All MAGs from *Ca*. Opimibacter, *Ca*. B. algidus, and *Ca*. P. calidus had all genes (*ppC, gltA, acnA, icd*) for degradation of phosphoenolpyruvate to 2-oxoglutarate, which is part of mixed acid fermentation (Friesen, 1988). In addition, the *adh* gene for acetaldehyde degradation to ethanol was present in all MAGs from *Ca*. Opimibacter, *Ca*. Vicinibacter, and *Ca*. D. haderslevense. Overall, 30 MAGs encoded alanine dehydrogenase (*ald*) for reduction of pyruvate to alanine. The reaction is reversible and has been reported to play an important role in redox balance maintenance during the fermentative growth and could additionally facilitate anaerobic growth or alanine use (Ward et al., 2000). Although all 5 genera have genes which, to some extent, may support anaerobic fermentation, it does not appear to be a key function.

Denitrification is important in wastewater treatment, converting nitrate (NO${}_{3}^{-}$) to dinitrogen gas (N_2_) (von Sperling, 2007). However, none of the MAGs analyzed were predicted to be full denitrifiers as they all lacked genes for dissimilatory nitrate reductase (*napAB, narGHI*), and other genes involved in the denitrification pathway were sporadically encoded by the different species (Figure 5). Genes for nitrite (NO${}_{2}^{-}$) reduction to nitric oxide (NO) (*nirK* or *nirS*) were predicted in nine MAGs from *Ca*. B. algidus, and NO reduction to nitrous oxide (N_2_O) (*norBC*) was predicted in 11 MAGs from *Ca*. O. iunctus (1/1), *Ca*. B. algidus (9/11), and *Ca*. P. calidus (1/8). Furthermore, the *norB* gene was annotated by EnrichM in 11 MAGs from *Ca*. D. haderslevense, *Ca*. Vicinibacter, and *Ca*. Opimibacter, while *norC* was present only in one MAG (Figure 5). Based on MicroScope annotation, in 10 of these 11 MAGs (Figure 5), the quinol-dependent nitric-oxide reductase gene (*norZ*) was predicted. NorZ can be found in many denitrifying organisms and various pathogenic species which lack the full denitrifying process. For those organisms, NorZ seems to be used for a defense, rather than energy conservation (Hendriks et al., 2000). The *nosZ* gene (K00376) was found in 23 MAGs representing *Ca*. P. calidus (8/8), *Ca*. Opimibacter (2/3), *Ca*. D. haderslevense (4/6), and *Ca*. Vicinibacter (9/10) (Figure 5), indicating a possible reduction of nitrous oxide (N_2_O) to N_2_. Potential usage of nitrous oxide as an electron acceptor was expected as the *nosZ* was previously predicted in five genomes from Bacteroidota (*Bacteroidetes bacterium* OLB8, OLB9, OLB10, OLB1, OLB12) (Speth et al., 2016). Overall, the use of oxidized nitrogen compounds in anaerobic respiration is hypothesized to be uncommon in *Saprospiraceae*, but perhaps some of them can be important as non-denitrifying nitrous oxide oxidizers (Ren et al., 2019). Nitrate/nitrite transporter, *NarK* gene (Noji et al., 1989), was not present in any of the genera, and multiple ABC transporter systems, which could be used for the translocation of many substrates across the membrane, including nitrogen-related compounds (Moir and Wood, 2001), were found in all five genera with a high copy number (Supplementary Data File 3).

Uptake and removal of phosphate are a key process among the PAOs and common in WWTPs with enhanced biological phosphorus removal (Nielsen et al., 2019). While no members of *Saprospiraceae* have previously been suggested to be PAOs, the full set of known genes encoding polyphosphate accumulation (*pit, pstABCS, phoU, ppk*) was predicted in all MAGs from *Ca*. D. haderslevense, *Ca*. Vicinibacter, *Ca*. Opimibacter, and *Ca*. P. calidus (Figure 5). In particular, the pit transporter gene (*pit*) for phosphate transport is suggested to be a main indicator for the PAO bacteria (Nielsen et al., 2019), suggesting these species could be potential PAOs.

Most PAOs accumulate PHA and/or glycogen as an intracellular carbon and energy source under dynamic oxic–anoxic conditions (Akbari et al., 2021; Petriglieri et al., 2021b). Previous studies of *Saprospiraceae* have not found PHA accumulation (Xia et al., 2008), but the genes (*PhaABC*) for PHA accumulation were predicted in 14 MAGs from the genera *Ca*. D. haderslevense and *Ca*. Vicinibacter (Figure 5), suggesting possible PHA accumulation and PHA usage. The full gene set for glycogen biosynthesis (*glgABC*) was encoded in 15 MAGs from *Ca*. Opimibacter, *Ca*. P. Validus, and *Ca*. B. algidus (Figure 5). Even though the well-known glycogen degradation pathway (M00855) was not present in *Ca*. Opimibacter, *Ca*. P. calidus, and *Ca*. B. algidus, all of them had the alpha-amylase (*AMY1A*) gene (Figure 5), which may be used for glycogen/starch degradation to dextrin and maltose (Janecek, 1997). Taken together, none of the MAGs investigated encoded both PHA and glycogen accumulation, suggesting they do not follow the conventional PAO metabolism.

### *In situ* Characterization of the Bacteria of Interest

**Figure 6:**
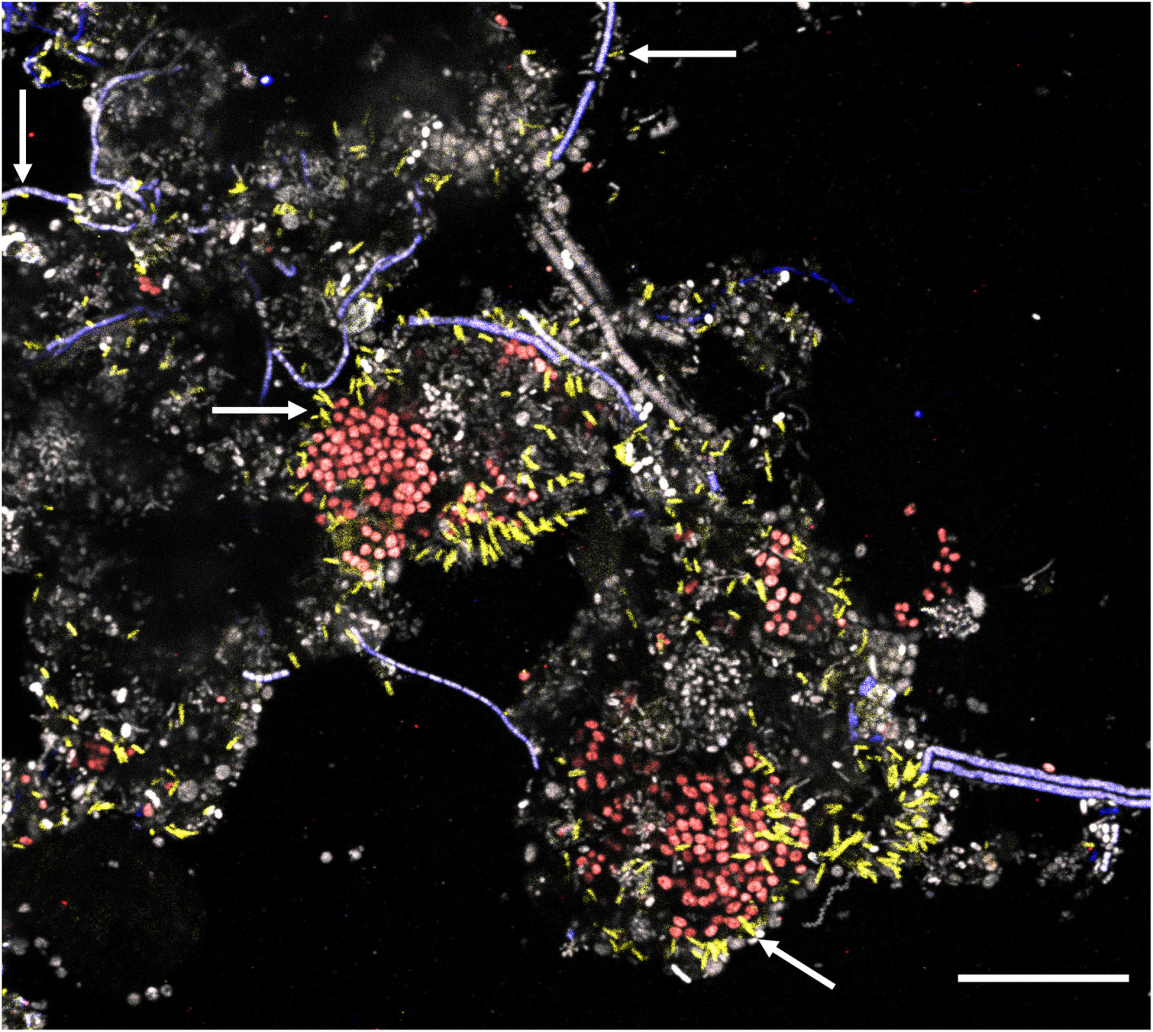
Multicolor FISH micrograph of *Ca*. Vicinibacter (yellow, G65-705) attached to *Ca*. Accumulibacter aalborgensis (red, Acc470 ref for that probe) and some to Chloroflexi filaments (blue, CFXmix ref for that probe). All other bacteria are gray. Scale bar is 20 μm.

New FISH probes were applied to full-scale activated sludge biomass to determine the morphology of the novel genera. Samples exclusively containing target species (based on amplicon sequencing) were selected to visualize individual species. All species were mostly present deep inside the flocs, usually as single cells with variations in size (Table 1). *Ca*. B. algidus was occasionally found as microcolonies. Although previous studies found some members of *Saprospiraceae* are epiphytic bacteria (Xia et al., 2008; McIlroy and Nielsen, 2014), only a few cells from *Ca*. Opimibacter, *Ca*. B. algidus, *Ca*. P. calidus, and *Ca*. D. haderslevense were attached to filamentous bacteria. Interestingly, members of *Ca*. Vicinibacter were attached to clusters of the PAO *Ca*. Accumulibacter aalborgensis and to a few filaments from the phylum Chloroflexota (Figure 6), indicating possible symbiotic relationships between these bacteria. This needs, however, to be verified by more detailed studies.

**Table 1:** Summary table of morphology and presence of intracellular storage polymers detected by FISH and FISH–Raman microspectroscopy in the *Saprospiraceae* species of interest.

**Target**	**Probe**	**Size, μm (wide × length)**	**PHA**	**Glycogen**	**poly-P**
*Ca*. Brachybacter algidus	OLB8-445	0.4–0.6 ×1–2	–	+	–
*Ca*. Parvibacillus calidus	OLB8-445	0.4–0.5 ×1.3–2	–	+	–
*Ca*. Opimibacter	G17-398	0.4–0.6 × 1.1–1.9	–	+	–
*Ca*. Defluviibacterium haderslevense	G65-705	0.4–0.5 × 2–3.2	+	–	–
*Ca*. Vicinibacter	G65-705	0.5–0.6 × 1.5–2.5	+	–	–

To confirm key physiological features of the novel *Saprospiraceae* genera, Raman microspectroscopy was performed in combination with the FISH. The species from the five genera showed *in situ* presence of peaks associated with common biological components, such as phenylalanine, nucleic acids, and lipids (Supplementary Figure S6). We did not detect poly-P in any of the genera, despite their potential based on the MAG annotation. Glycogen was detected in *Ca*. B. algidus, *Ca*. P. calidus, and *Ca*. Opimibacter (Table 1 and Supplementary Figure S6), whereas *Ca*. D. haderslevense and *Ca*. Vicinibacter had a peak indicative of the storage polymer PHA (Table 1 and Supplementary Figure S6), agreeing with the metabolic annotation. These results indicate that they are not PAOs, but the storage polymers glycogen or PHA may be important under dynamic substrate conditions.

## Ecological Role of *Saprospiraceae* in WWTPs

*Saprospiraceae* is a diverse family comprising 162 genera as assessed by our comprehensive global analysis. However, as only a few genera were present and abundant in most WWTPs globally, we have mainly focused on these, although many other genera and species could be abundant in a few plants across the world. The huge variation from plant to plant may be due to the high impact of immigration from different source communities to the WWTPs (Dottorini et al., 2021). The highly abundant former genus OLB8 was found in different WWTP designs, which comprises two different genera, *Ca*. Brachybacter and *Ca*. Parvibacillus, based on ANI. *Ca*. Brachybacter and *Ca*. Parvibacillus were mainly present in conventional nutrient removal plants and in deammonification plants, respectively.

The only abundant *Saprospiraceae* isolate from WWTPs is *Haliscomenobacter*, which is described as strictly aerobic heterotrophic filamentous bacteria. Similar to *Ca*. Epiflobacter, *Haliscomenobacter* has the ability to degrade polysaccharides and amino acids with the proposed ability to accumulate carbohydrate storage polymers but not PHA (van Veen et al., 1973; Xia et al., 2008). Our study showed that all analyzed genera have the potential to degrade different polysaccharides such as starch and many amino acids and may grow under both oxic and anoxic conditions. Some members from *Saprospiraceae* (*Ca*. Epiflobacter) are also known to be epiphytic bacteria attached to filamentous bacteria and may have a symbiotic relationship. However, only *Ca*. Vicinibacter was found to be attached in some WWTPs to *Ca*. Accumulibacter and some filamentous bacteria from the Chloroflexota phylum.

As bacteria from *Saprospiraceae* are often found in WWTPs designed for nutrient removal, it is assumed that they are involved in the removal of nitrogen and phosphorus. Specifically, *Ca*. Parvibacillus was abundantly present in deammonification plants, which are characterized by high temperature (20.1–30.0°C) and high ammonium concentration. However, none of the genera analyzed were denitrifiers as nitrate and nitrite reductases were not encoded or encoded in only a few of the MAGs, respectively. Only *Ca*. Brachybacter algidus had a gene set for reduction of NO${}_{2}^{-}$ to N_2_O. Most MAGs encoded nitrous oxide reductase, indicating that they might act as non-denitrifying nitrous oxide reducers, but further experiments are needed to confirm this.

Some genera had a potential to be PAOs, but none of them had the canonical pathway known from *Ca*. Accumulibacter and *Ca*. Dechloromonas with the dynamic accumulation of poly-P, PHA, and glycogen (Petriglieri et al., 2021a,b). Although the genera had the potential to accumulate poly-P, this was not observed in our *in situ* studies. Physiological experiments are required to find or confirm PAO metabolism as the genes are indicative but not conclusive. As predicted, *Ca*. D. haderslevense and *Ca*. Vicinibacter accumulated PHA, but not glycogen. *Ca*. Opimibacter, *Ca*. Brachybacter, and *Ca*. Parvibacillus calidus all stored glycogen, but not PHA, so they all seem well adapted to dynamic oxic–anoxic conditions but seemed not to be directly involved in the biological P removal.

Our study provides a deeper insight into a very diverse *Saprospiraceae* family through *in situ* studies and genomic information that could also be used for future research, for example, using metatranscriptomics, in order to fully understand and confirm this family's involvement in different processes of wastewater treatment.

## Etymology

Description of “*Candidatus* Brachybacter algidus” gen. nov. sp. nov. (OLB8 midas_s_29) “*Candidatus* Brachybacter algidus,” (bra.chi.bac'ter, G. m. adj. *brachis*, small; N.L. m. n. *bacteri*, rod-shaped bacterium; N.L. m. n. *Brachybacter*, indicating a small rod-shaped bacterium; al'gi.dus, L. m. adj. *algidus*, indicating the highest abundance of this microorganism in colder climates). This taxon was represented by the MAG Hade_18-Q3-R52-61_MAXAC.021. The complete protologue can be found in Supplementary Table S6.

Description of “*Candidatus* Parvibacillus calidus” gen. nov. sp. nov. (OLB8 midas_s_3279) “*Candidatus* Parvibacillus calidus,” (par.vi.ba.cil'lus, L. m. adj. *parvus*, small; N.L. m. n. *bacillus*, rod-shaped bacterium; N.L. m. n. *parvibacillus*, indicating a small rod-shaped bacterium; ca'li.dus, L. m. adj. *calidus*, indicating the highest abundance of this microorganism in warmer climates). This taxon was represented by GCA_013414645.1. The complete protologue can be found in Supplementary Table S7.

Description of “*Candidatus* Defluviibacterium haderslevense” gen. nov. sp. nov. (midas_g_65 midas_s_65) “*Candidatus* Defluviibacterium haderslevense,” (de.flu.vi.i.bac.te'rium, L. n. n. *defluvium*, sewage; N.L. m. n. *bacterium*, rod-shaped bacterium; N.L. m. n. *defluviibacterium*, indicating a rod-shaped bacterium found in sewage; ha.der.sle.ven'sis, N. L. fem. adj. haderslevense pertaining to the city of Haderslev, where the sample was obtained from which the MAG was produced). This taxon was represented by the MAG Hade_18-Q3-R52-61_BAT3C.141. The complete protologue can be found in Supplementary Table S8.

Description of “*Candidatus* Vicinibacter” gen. nov. (midas_g_65 midas_s_177) “*Candidatus* Vicinibacter,” (vi.ci.ni.bac'ter, L. m. adj. *vicinus*, close; N.L. m. n. *bacter*, rod-shaped bacterium; N.L. m. n. *vicinibacter*, indicating rod-shaped bacteria often attached to other bacterial clusters). Description of “*Candidatus* Vicinibacter affinis” sp. nov. “*Candidatus* Vicinibacter affinis,” (af.fi'nis L. m. adj. *affinis*, indicating the close phylogenetic relationship with the other *Candidatus* Vicinibacter species). This taxon was represented by the MAG EsbW_18-Q3-R4-48_MAXAC.074. The complete protologue can be found in Supplementary Table S10. Description of “*Candidatus* Vicinibacter proximus” sp. nov. “*Candidatus* Vicinibacter proximus,” (pro'xi.mus, L. m. adj. *proximus*, indicating the close phylogenetic relationship with the other *Candidatus* Vicinibacter species). This taxon was represented by the MAG Fred_18-Q3-R57-64_BAT3C.331. The complete protologue can be found in Supplementary Table S9.

Description of “*Candidatus* Opimibacter” gen. nov. (midas_g_17 midas_s_17) “*Candidatus* Opimibacter,” (o.pi.mi.bac'ter, L. m. adj. *opimus*, fat; N.L. m. n. *bacter*, rod-shaped bacterium; N.L. m. n. *opimibacter*, indicating a fat rod-shaped bacterium). Description of “*Candidatus* Opimibacter skivensis” sp. nov. “*Candidatus* Opimibacter skivensis,” (ski.ven'sis, N.L. m. adj. *skivensis*, indicating the location of Skive, from where the MAG was obtained). This taxon was represented by the MAG Skiv_18-Q3-R9-52_BAT3C.266. The complete protologue can be found in Supplementary Table S11. Description of “*Candidatus* Opimibacter iunctus” sp. nov. “*Candidatus* Opimibacter iunctus,” (iun'ctus, L. m. adj. *iunctus*, indicating the close phylogenetic relationship with *Candidatus* Opimibacter skivensis). This taxon was represented by the MAG Hjor_18-Q3-R7-51_BAT3C.56. The complete protologue can be found in Supplementary Table S12.

## Data Availability Statement

The original contributions presented in the study are included in the article/Supplementary Material, further inquiries can be directed to the corresponding author.

## Author Contributions

ZK and PN designed the study and wrote the manuscript. FP and MN performed 16S rRNA gene phylogenetic analyses. MN designed the FISH probes. ZK performed the FISH probe optimization, imaging, and qFISH. FP performed the FISH–Raman analyses. ZK, LV, and CS performed the phylogenomic analysis and metabolic reconstruction. ZK and MD performed amplicon sequencing bioinformatic analysis. All authors reviewed and approved the final manuscript.

## Funding

This project was funded by the Villum Foundation (Dark Matter grant13351) and Aalborg University.

## Conflict of Interest

The authors declare that the research was conducted in the absence of any commercial or financial relationships that could be construed as a potential conflict of interest.

## Publisher's Note

All claims expressed in this article are solely those of the authors and do not necessarily represent those of their affiliated organizations, or those of the publisher, the editors and the reviewers. Any product that may be evaluated in this article, or claim that may be made by its manufacturer, is not guaranteed or endorsed by the publisher.
