# Supplementary material for: Diversity and Ecophysiology of the Genus OLB8 and Other Abundant Uncultured Saprospiraceae Genera in Global Wastewater Treatment Systems
Source: Front Microbiol. 2022 Jul 8;13:917553. doi: 10.3389/fmicb.2022.917553 (PMC9304909; doi:10.3389/fmicb.2022.917553)
Supplement: Supplementary file 5 [file Data_Sheet_5.pdf]

Supplementary Material

Figure S1. Global average relative read abundance across global WWTPs of top 10 most abundant phyla.

Figure S2. Time series of average abundance of OLB8 midas_s_3279 in an anammox reactor over the period of 6 months.

Figure S3. FISH micrograph of genus-specific FISH probes for the targeted genera of the *Saprospiraceae.*

Figure S4. Scatterplot showing abundance distribution of *Saprospiraceae* based on V4 and V1-V3 primers.

Figure S5. ANIb of the MAGs and genomes belonging to the *Ca.* D. haderslevense, *Ca.* Vicinibacter, *Ca.* Opimibacter, *Ca.* B. algidus, and *Ca.* P. calidus.

Figure S6. Raman spectra of the genera *Ca.* Opimibacter, *Ca.* Parvibacillus, and *Ca.* Vicinibacter, highlighting the differences in storage polymers content.

Table S1. FISH probes used in this study.

Table S2. List of countries belonging to different climate zones.

Table S3. Exact p-values of Kursak-Wallis statistical test.

Table S4. qFISH results.

Table S5. Number of MAGs representing different MiDAS-defined species from the *Saprospiraceae.*

Table S6. Protologues table for *Ca.* Brachybacter algidus.

Table S7. Protologues table for *Ca.* Parvibacillus calidus.

Table S8. Protologues table for *Ca.* Defluviibacterium haderslevense.

Table S9. Protologues table for *Ca.* Vicinibacter proximus.

Table S10. Protologues table for *Ca.* Vicinibacter affinis.

Table S11. Protologues table for *Ca.* Opimibacter skivensis.

Table S12. Protologues table for *Ca.* Opimibacter iunctus.

Data S1. MAG statistics. Including CDS regions, NCBI accession and taxonomy information (separate file).

Data S2. 16S rRNA gene ANIb table for MAGs used in this study (separate file).

Data S3. KOs and copy number of genes present in MAGs, mentioned in the text and used for the Figure 5 (separate file).

Data S4. KEGG modules identified with 100% completeness in the *Ca*. Opimibacter, *Ca*. Defluviibacterium, *Ca*. Vicinibacter, *Ca*. Brachybacter and *Ca*. Parvibacillus (separate file).

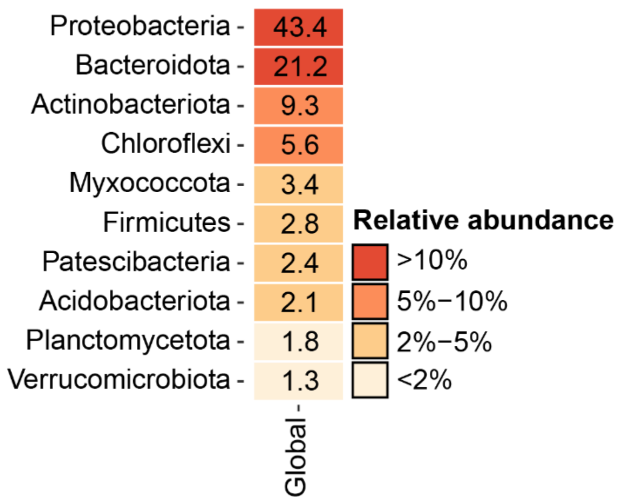

Figure S1. Global average relative abundance across global WWTPs of top 10 most abundant phyla.

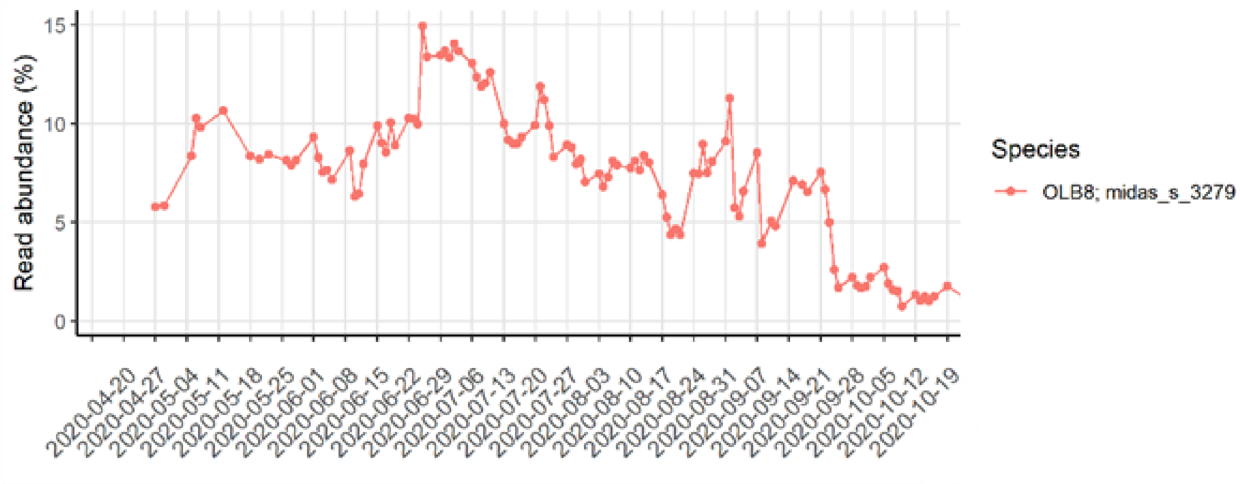

Figure S2. Time series of average read abundance of OLB8 midas_s_3279 (*Ca.* Parvibacillus calidus) in an anammox reactor over the period of 6 months.

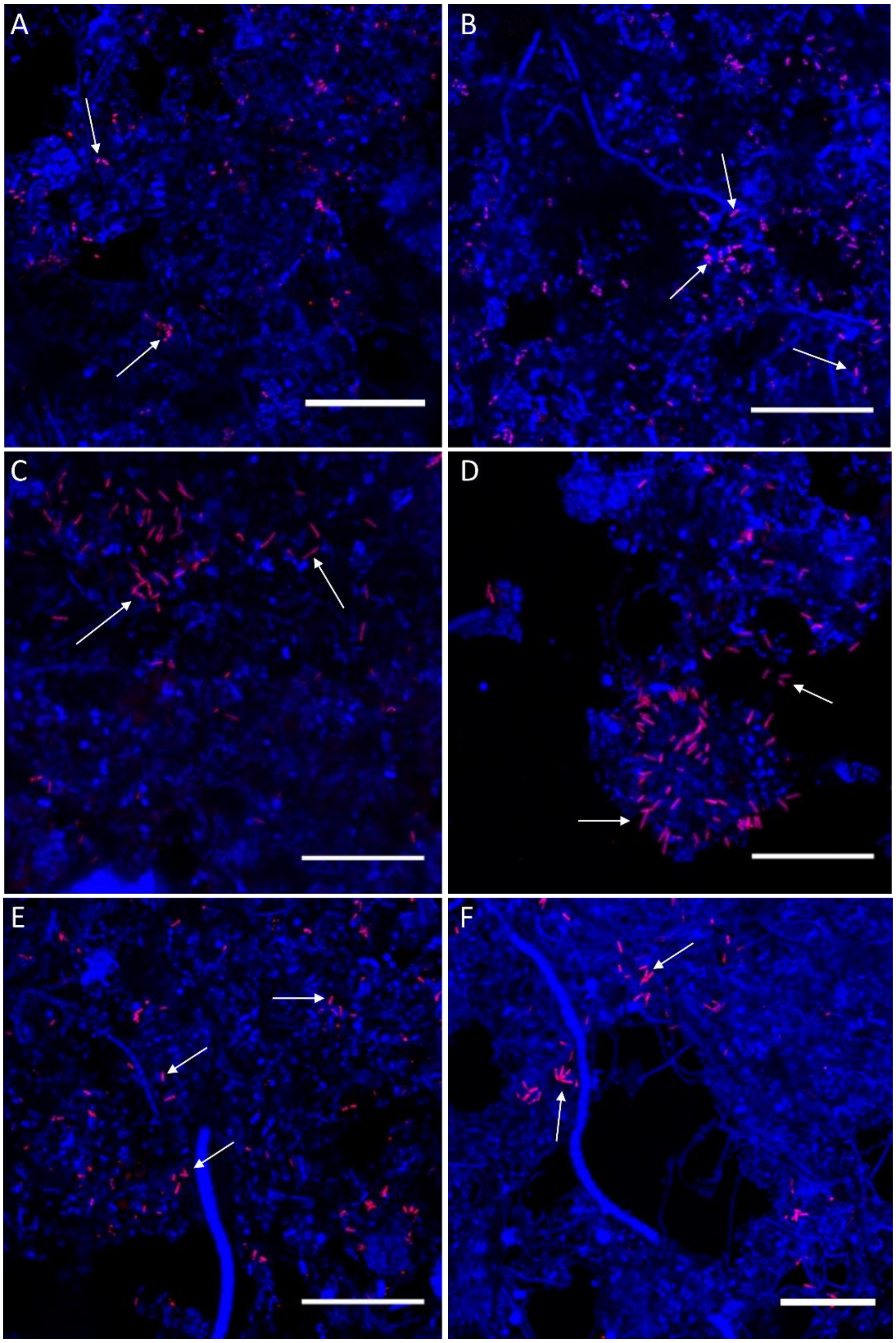

Figure S3. FISH micrograph of genus-specific FISH probes for the targeted genera of the *Saprospiraceae*. In all pictures bacteria targeted by the specific probe are in magenta, and all other bacteria targeted by EUBmix are in blue. **(A)** *Ca.* Brachybacter algidus (OLB8 midas_s_29); **(B)** – *Ca.* Parvibacillus algidus (OLB8 midas_s_3729); **(C)** – *Ca.* Defluviibacterium haderslevense (midas_g_65 midas_s_65); **(D)** – *Ca.* Vicinibacter (midas_g_65 midas_s_177); **(E)** – *Ca.* Opimibacter (midas_g_17 midas_s_s17); **(F)** – the *Saprospiraceae* (targeted by SAP317 probe). Scale bar in all images: 20 µm.

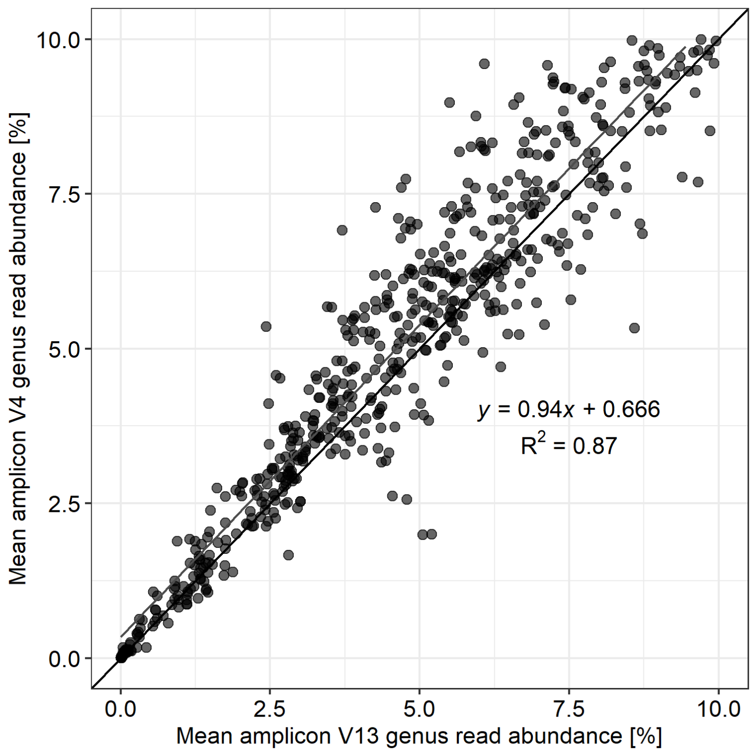

Figure S4. Scatterplot showing abundance distribution of the *Saprospiraceae* based on V4 and V1-V3 primers.

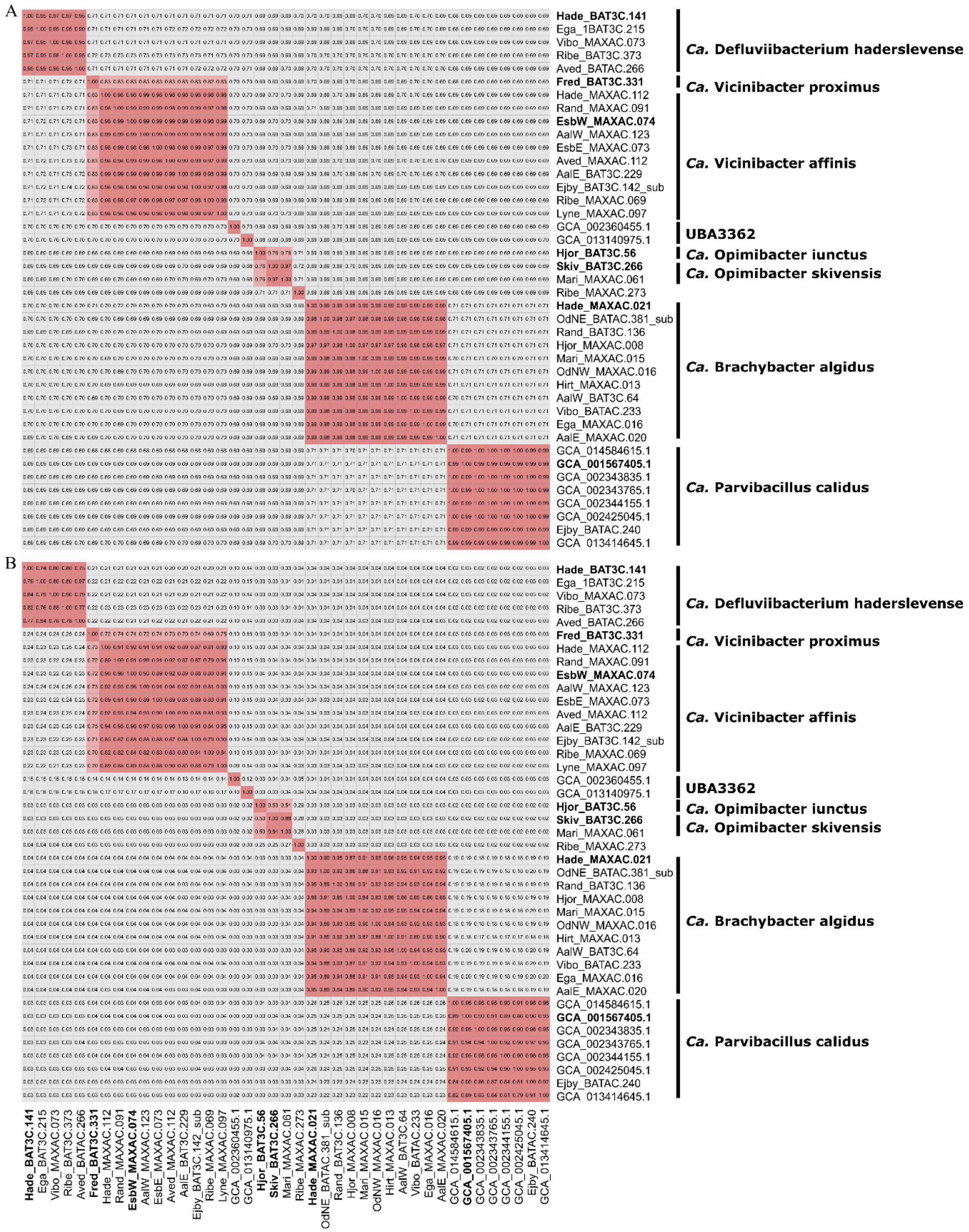

Figure S5. ANIb of the MAGs and genomes belonging to the *Ca.* D. haderslevense, *Ca.* Vicinibacter, *Ca.* Opimibacter, *Ca.* B. algidus and *Ca.* P. calidus. **A** – ANIb; **B** – Alignment coverage of ANIb. Representative MAGs of each species are in bold.

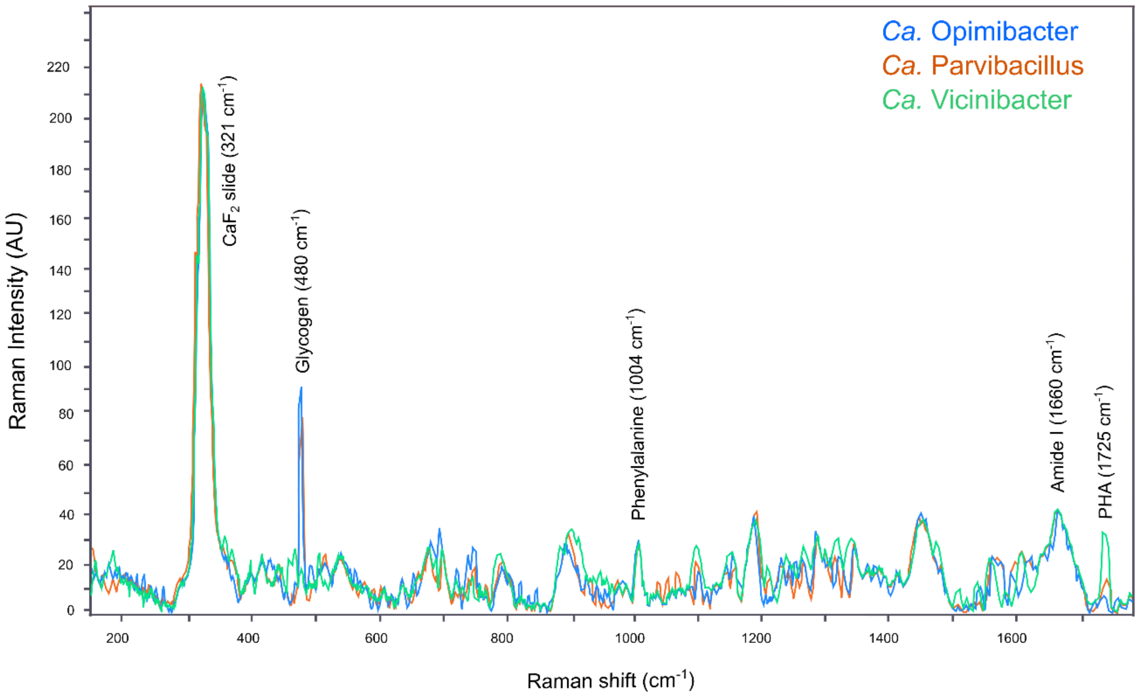

Figure S6. Raman spectra of species from the genera *Ca.* Opimibacter, *Ca.* Parvibacillus, and *Ca.* Vicinibacter, highlighting the differences in storage polymers content. Peaks for phenylalanine and amide in linkages of proteins are specific markers for biological material. Peaks for glycogen, polyphosphate, and PHA are indicating the presence of the PAO-specific storage polymers. Spectra are calculated as average of 100 FISH-defined cells. AU, arbitrary units.

Table S1. FISH probes used in this study.

Probe	*E. coli* pos.	Target group	Coverage *	Non-target hits	Sequence (5'-3')	[FA]%**	Reference
**SAP309**	**309-327**	***Saprospiraceae* family**	**1989/2544**		**TCT CAG TAC CCG TGT GGG**	**25**	**Schauer and Hahn, 2005**
**SAP317**	**317-337**	***Saprospiraceae* family**	**2511/2544**	**0**	**GGG TCC GTG TCT CAG TAC CC**	**30**	**This study**
SAP317_C1	317-337	Competitor probe for SAP317	NA	NA	GGG TCC GTM TCT CAG TAC CC	NA	This study
**OLB8-455**	**445-466**	**OLB8**	**50/53**	**3**	**TCA GTC CAG GAT AAA TCC CGT**	**50**	**This study**
**G65-706**	**706-730**	**midas_g_65**	**17/17**	**0**	**TTC GCA ATC GAT GTT CCA TGG CAT**	**45**	**This study**
G65-706_C1	706-730	Competitor for probe g65-706	NA	NA	TTC GCA ATC GAT GTT CCA TGA CAT	NA	This study
G65-706_C2	706-730	Competitor for probe g65-706	NA	NA	TTC GCA ATC GAT GTT CTA TGG CAT	NA	This study
**G17-398**	**398-417**	**midas_g_17**	**105/105**	**0**	**CTT CTT CCT GCA CGC TGG A**	**50**	**This study**
G17-398_C1	398-417	Competitor probe for g17-398	NA	NA	CTT CTT CCT GCA CGC GGG A	NA	This study
G17-398_C2	398-416	Competitor probe for g17-398	NA	NA	CGT CTT CCT GCA CGC TGG	NA	This study
G17-398_H1	398-428	Helper probe for g17-398	NA	NA	CTG ATA AAA GCA GTT TAC AAC CCA TAG GGC	NA	This study
G17-398_H2	398-417	Helper probe for g17-398	NA	NA	CTG GAT CAG CCT TKC GGC C	NA	This study

* Taxonomy and coverage of groups is defined as in the MiDAS 4 database. Values given as group hits/group totals; ** Recommended optimal formamide concentration for use in FISH hybridization; N/A – not applicable.

Table S2. List of countries belonging to different climate zones.

Abbreviation	Climate	Countries
**A**	Tropical/mesothermal	India, Malaysia, Philippines, Singapore
**B**	Dry (desert and semi-arid)	Argentina, Australia, Canada, China, Israel, India, Mexico, Saudi Arabia, Spain, USA
**C**	Temperate/mesothermal	Argentina, Australia, Austria, Belgium, China, Cyprus, Czech Republic, Denmark, Germany, Hong Kong, Israel, Italy, Netherlands, Norway, Portugal, Poland, Spain, South Africa, Switzerland, Sweden, UK, USA, Uruguay
**D**	Continental/microthermal	Canada, China, Finland, Norway, Poland, South Africa, Sweden, USA
**E**	Polar	Switzerland

Table S3. Exact p-values for Kruskal-Wallis statistical test.

	**Climate zones**	**Temperature range**	**Process type**	**Fraction of industrial wastewater**
**midas_s_17**	2.2e-16	2.2e-16	2.2e-16	3.063e-16
**midas_s_177**	0.0000000004149	6.536e-12	2.2e-16	1.553e-12
**midas_s_29**	2.2e-16	0.000000007198	2.2e-16	0.0000000002588
**midas_s_3279**	2.2e-16	0.001487	0.0002022	0.002376
**midas_s_65**	2.945e-14	2.2e-16	2.2e-16	2.2e-16

Table S4. qFISH results.

WWTP	Sample date	Abundance (%)	
		**Sequencing**	**qFISH**
**OLB8 (MK04-455) (*Ca.* B. algidus & *Ca.* P. calidus)**			
Esbjerg East, Denmark	2011 February	3	0.8 ± 0.3
Viborg, Denmark	2011 February	4.5	1.5 ± 0.9
Skive, Denmark	2017 February	3.9	0.9 ± 0.3
**Midas_g_17 (G17-398) (*Ca.* Opimibacter)**			
Esbjerg East, Denmark	2006 May	1.3	0.7 ± 0.6
Ribe, Denmark	2016 August	3.1	<1
Odense East, Denmark	2018 August	1.6	<1
**Migas_g_65 (G65-705) (*Ca.* D. haderslevense & *Ca.* Vicinibacter)**			
Skive, Denmark	2007 October	2.1	1.4 ± 0.4
Ribe, Denmark	2015 February	1.6	0.8 ± 0.5
Fredericia, Denmark	2017 May	4.1	2.3 ± 1

Table S5. Numbers of MAGs representing different MiDAS-defined species from the *Saprospiraceae*.

MiDAS genus name	MiDAS species name	No. of MAGs
**OLB8**	midas_s_29	11
	midas_s_3279	1
**midas_g_65**	midas_s_65	5
	midas_s_177	11
**midas_g_17**	midas_s_17	3
	midas_s_632	1

Table S6. Protologues table for *Ca.* Brachybacter algidus.

Species name	*Candidatus* Brachybacter algidus
Genus name	*Candidatus* Brachybacter
Specific epithet	algidus
Type species of the genus	*Candidatus* Brachybacter algidus
Genus status	Candidatus
Species etymology	"*Candidatus* Brachybacter algidus", (bra.chi.bac´ter, G. m. adj. brachis, small; N.L. m. n. bacteri, rod-shaped bacterium; N.L. m. n. Brachybacter, indicating a small rod-shaped bacterium; al´gi.dus, L. m. adj. algidus, indicating the highest abundance of this microorganism in colder climates).
Species status	sp. nov.
Designation of the type MAG	GCA_016711705.1
MAG/SAG accession number	GCA_016711705.1
Genome status	High-quality draft
Genome size	4694034
GC mol %	36.6
Country of origin	Denmark
Region of origin	Haderslev
Source of sample	Full-scale enriched biological phosphorus removal wastewater treatment plant
Geographical location	Haderslev
Latitude	55.249786
Longitude	9.508609
Depth	N/A
Altitude	N/A
Temperature of the sample	Mesophilic
pH of the sample	N/A
Relationship to oxygen	facultative anaerobe
Energy metabolism	Likely utilizes a range of substrates including amino acids and polysaccharides.
Assembly	1 sample
Sequencing technology	Oxford Nanopore and Illumina Hiseq X
Binning software used	MaxBin2
Assembly software used	CANU v1.8
Habitat	Full-scale enriched biological phosphorus removal wastewater treatment plant
Miscellaneous, extraordinary features relevant for the description	Rod shaped cells 0.5±0.1 x 1-2 µm (diameter x length), usually found as a single cell deep inside the floc, sometimes found in a few cell bundles.

Table S7. Protologues table for *Ca.* Parvibacillus calidus.

Species name	*Candidatus* Parvibacillus calidus
Genus name	*Candidatus* Parvibacillus
Specific epithet	calidus
Type species of the genus	*Candidatus* Parvibacillus calidus
Genus status	Candidatus
Species etymology	"*Candidatus* Parvibacillus calidus", (par.vi.ba.cil´lus, L. m. adj. *parvus*, small; N.L. m. n. *bacillus*, rod-shaped bacterium; N.L. m. n. *parvibacillus*, indicating a small rod-shaped bacterium; ca´li.dus, L. m. adj. *calidus*, indicating the highest abundance of this microorganism in warmer climates).
Species status	sp. nov.
Designation of the type MAG	GCA_013414645.1
MAG/SAG accession number	GCA_013414645.1
Genome status	High-quality draft
Genome size	3913768
GC mol %	41.3
Country of origin	Singapore
Region of origin	Jurong West
Source of sample	Activated sludge
Geographical location	Singapore: Jurong West
Latitude	1.344722
Longitude	103.681389
Depth	N/A
Altitude	N/A
Temperature of the sample	N/A
pH of the sample	N/A
Relationship to oxygen	N/A
Energy metabolism	Likely utilizes a range of substrates including amino acids and polysaccharides.
Assembly	1 sample
Sequencing technology	Oxford Nanopore
Binning software used	MetaBAT2
Assembly software used	CANU v1.8
Habitat	Continuous culture bioreactor
Miscellaneous, extraordinary features relevant for the description	Rod shaped cells 0.4-0.5 x 1.3-2 μm (diameter x length), usually found as a single cell deep inside the floc.

Table S8. Protologues table for *Ca.* Defluviibacterium haderslevense.

Species name	*Candidatus* Defluviibacterium haderslevense
Genus name	*Candidatus* Defluviibacterium
Specific epithet	haderslevense
Type species of the genus	*Candidatus* Defluviibacterium haderslevense
Genus status	Candidatus
Species etymology	Description of *'Candidatus* Defluviibacterium haderslevense' gen. nov. sp. nov. (midas_g_65 midas_s_65) "*Candidatus* Defluviibacterium haderslevense", (de.flu.vi.i.bac.te´rium, L. n. n. *defluvium,* sewage; N.L. m. n. *bacterium*, rod-shaped bacterium; N.L. m. n. *defluviibacterium*, indicating a rod-shaped bacterium found in sewage; ha.der.sle.ven'sis, N. L. fem. adj. haderslevense pertaining to the city of Haderslev, where the sample was obtained from which the MAG was produced).
Species status	sp. nov.
Designation of the type MAG	GCA_016712225.1
MAG/SAG accession number	GCA_016712225.1
Genome status	High-quality draft
Genome size	52401
GC mol %	33.1
Country of origin	Denmark
Region of origin	Haderslev
Source of sample	Full-scale enriched biological phosphorus removal wastewater treatment plant
Geographical location	Haderslev
Latitude	55.249786
Longitude	9.508609
Depth	N/A
Altitude	N/A
Temperature of the sample	Mesophilic
pH of the sample	N/A
Relationship to oxygen	facultative anaerobe
Energy metabolism	Likely utilizes a range of substrates including amino acids and polysaccharides.
Assembly	1 sample
Sequencing technology	Oxford Nanopore and Illumina Hiseq X
Binning software used	MetaBAT2
Assembly software used	CANU v1.8
Habitat	Full-scale enriched biological phosphorus removal wastewater treatment plant
Miscellaneous, extraordinary features relevant for the description	Rod shaped cells 0.4-0.5 x 2-3.2 μm (diameter x length), usually found as a single cells deep inside the floc.

Table S9. Protologues table for *Ca.* Vicinibacter proximus.

Species name	*Candidatus* Vicinibacter proximus
Genus name	*Candidatus* Vicinibacter
Specific epithet	proximus
Type species of the genus	*Candidatus* Vicinibacter proximus
Genus status	Candidatus
Species etymology	"*Candidatus* Vicinibacter", (vi.ci.ni.bac´ter, L. m. adj. *vicinus,* close; N.L. m. n. *bacter*, rod-shaped bacterium; N.L. m. n. *vicinibacter,* indicating rod-shaped bacteria often attached to other bacterial clusters). *'Candidatus* Vicinibacter proximus' sp. nov. "*Candidatus* Vicinibacter proximus", (pro´xi.mus, L. m. adj. *proximus*, indicating the close phylogenetic relationship with the other *Candidatus* Vicinibacter species).
Species status	sp. nov.
Designation of the type MAG	GCA_016714365.1
MAG/SAG accession number	GCA_016713905.1
Genome status	High-quality draft
Genome size	4496903
GC mol %	37
Country of origin	Denmark
Region of origin	Fredericia
Source of sample	Full-scale enriched biological phosphorus removal wastewater treatment plant
Geographical location	Fredericia
Latitude	55.552368
Longitude	9.720404
Depth	N/A
Altitude	N/A
Temperature of the sample	Mesophilic
pH of the sample	N/A
Relationship to oxygen	facultative anaerobe
Energy metabolism	Likely utilizes a range of substrates including sugars, amino acids and polysaccharides.
Assembly	1 sample
Sequencing technology	Oxford Nanopore and Illumina Hiseq X
Binning software used	MetaBAT2
Assembly software used	CANU v1.8
Habitat	Full-scale enriched biological phosphorus removal wastewater treatment plant
Miscellaneous, extraordinary features relevant for the description	Rod shaped cells 0.5-0.6 x 1.5-2.5 µm (diameter x length), found either as a single cell deep inside the flocs or attached to other bacteria.

Table S10. Protologues table for *Ca.* Vicinibacter affinis.

Species name	*Candidatus* Vicinibacter affinis
Genus name	*Candidatus* Vicinibacter
Specific epithet	affinis
Type species of the genus	*Candidatus* Vicinibacter affinis
Genus status	Candidatus
Species etymology	"*Candidatus* Vicinibacter", (vi.ci.ni.bac´ter, L. m. adj. *vicinus,* close; N.L. m. n. *bacter*, rod-shaped bacterium; N.L. m. n. *vicinibacter*, indicating rod-shaped bacteria often attached to other bacterial clusters). '*Candidatus* Vicinibacter affinis' sp. nov. "*Candidatus* Vicinibacter affinis", (af.fi´nis L. m. adj. *affinis*, indicating the close phylogenetic relationship with the other *Candidatus* Vicinibacter species).
Species status	sp. nov.
Designation of the type MAG	GCA_016714365.1
MAG/SAG accession number	GCA_016714365.1
Genome status	High-quality draft
Genome size	4661800
GC mol %	37.3
Country of origin	Denmark
Region of origin	Esbjerg West
Source of sample	Full-scale biological nutrient removal wastewater treatment plant
Geographical location	Esbjerg West
Latitude	55.488097
Longitude	8.430505
Depth	N/A
Altitude	N/A
Temperature of the sample	Mesophilic
pH of the sample	N/A
Relationship to oxygen	facultative anaerobe
Energy metabolism	Likely utilizes a range of substrates including sugars, amino acids and polysaccharides.
Assembly	1 sample
Sequencing technology	Oxford Nanopore and Illumina Hiseq X
Binning software used	MaxBin2
Assembly software used	CANU v1.8
Habitat	Full-scale biological nutrient removal wastewater treatment plant
Miscellaneous, extraordinary features relevant for the description	Rod shaped cells 0.5-0.6 x 1.5-2.5 μm (diameter x length), found either as a single cell deep inside the flocs or attached to other bacteria.

Table S11. Protologues table for *Ca.* Opimibacter skivensis.

Species name	*Candidatus* Opimibacter skivensis
Genus name	*Candidatus* Opimibacter
Specific epithet	skivensis
Type species of the genus	*Candidatus* Opimibacter skivensis
Genus status	Candidatus
Species etymology	"*Candidatus* Opimibacter", (o.pi.mi.bac´ter, L. m. adj. *opimus*, fat; N.L. m. n. *bacter*, rod-shaped bacterium; N.L. m. n. *opimibacter*, indicating a fat rod-shaped bacterium). Description of *'Candidatus* Opimibacter skivensis' sp. nov. "*Candidatus* Opimibacter skivensis", (ski.ven´sis, N.L. m. adj. *skivensis*, indicating the location of Skive, from where the MAG was obtained).
Species status	sp. nov.
Designation of the type MAG	GCA_016716665.1
MAG/SAG accession number	GCA_016721905.1
Genome status	High-quality draft
Genome size	5354596
GC mol %	44.9
Country of origin	Denmark
Region of origin	Skive
Source of sample	Full-scale enriched biological phosphorus removal wastewater treatment plant
Geographical location	Skive
Latitude	56.565132
Longitude	9.042158
Depth	N/A
Altitude	N/A
Temperature of the sample	Mesophilic
pH of the sample	N/A
Relationship to oxygen	facultative anaerobe
Energy metabolism	Likely utilizes a range of substrates including amino acids and polysaccharides.
Assembly	1 sample
Sequencing technology	Oxford Nanopore and Illumina Hiseq X
Binning software used	MetaBAT2
Assembly software used	CANU v1.8
Habitat	Full-scale enriched biological phosphorus removal wastewater treatment plant
Miscellaneous, extraordinary features relevant for the description	Rod shaped cells 0.5±0.1 x 1.5±0.4 µm (diameter x length), usually found as a single cell deep inside the floc.

Table S12. Protologues table for *Ca.* Opimibacter iunctus.

Species name	*Candidatus* Opimibacter iunctus
Genus name	*Candidatus* Opimibacter
Specific epithet	iunctus
Type species of the genus	*Candidatus* Opimibacter iunctus
Genus status	Candidatus
Species etymology	Description of *'Candidatus* Opimibacter' gen. nov. (midas_g_17 midas_s_17) "*Candidatus* Opimibacter", (o.pi.mi.bac´ter, L. m. adj. *opimus*, fat; N.L. m. n. *bacter*, rod-shaped bacterium; N.L. m. n. *opimibacter*, indicating a fat rod-shaped bacterium). Description of *'Candidatus* Opimibacter iunctus' sp. nov. "*Candidatus* Opimibacter iunctus", (iun´ctus, L. m. adj. *iunctus*, indicating the close phylogenetic relationship with *Candidatus* Opimibacter skivensis).
Species status	sp. nov.
Designation of the type MAG	GCA_016716665.1
MAG/SAG accession number	GCA_016716665.1
Genome status	High-quality draft
Genome size	4887894
GC mol %	46.4
Country of origin	Denmark
Region of origin	Hjørring
Source of sample	Full-scale enriched biological phosphorus removal wastewater treatment plant
Geographical location	Hjørring
Latitude	57.421265
Longitude	9.975411
Depth	N/A
Altitude	N/A
Temperature of the sample	Mesophilic
pH of the sample	N/A
Relationship to oxygen	facultative anaerobe
Energy metabolism	Likely utilizes a range of substrates including amino acids and polysaccharides.
Assembly	1 sample
Sequencing technology	Oxford Nanopore and Illumina Hiseq X
Binning software used	MetaBAT2
Assembly software used	CANU v1.8
Habitat	Full-scale enriched biological phosphorus removal wastewater treatment plant
Miscellaneous, extraordinary features relevant for the description	Rod shaped cells 0.5±0.1 x 1.5±0.4 µm (diameter x length), usually found as a single cell deep inside the floc.

References

Schauer, M., Hahn, M.W., 2005. Diversity and phylogenetic affiliations of morphologically conspicuous large filamentous bacteria occurring in the pelagic zones of a broad spectrum of freshwater habitats. Appl. Environ. Microbiol. 71, 1931–1940. https://doi.org/10.1128/AEM.71.4.1931-1940.2005
